# The Effects of Dietary Carotenoid Supplementation and Retinal Carotenoid Accumulation on Vision-Mediated Foraging in the House Finch

**DOI:** 10.1371/journal.pone.0021653

**Published:** 2011-06-29

**Authors:** Matthew B. Toomey, Kevin J. McGraw

**Affiliations:** School of Life Sciences, Arizona State University, Tempe, Arizona, United States of America; Lund University, Sweden

## Abstract

**Background:**

For many bird species, vision is the primary sensory modality used to locate and assess food items. The health and spectral sensitivities of the avian visual system are influenced by diet-derived carotenoid pigments that accumulate in the retina. Among wild House Finches (*Carpodacus mexicanus*), we have found that retinal carotenoid accumulation varies significantly among individuals and is related to dietary carotenoid intake. If diet-induced changes in retinal carotenoid accumulation alter spectral sensitivity, then they have the potential to affect visually mediated foraging performance.

**Methodology/Principal Findings:**

In two experiments, we measured foraging performance of house finches with dietarily manipulated retinal carotenoid levels. We tested each bird's ability to extract visually contrasting food items from a matrix of inedible distracters under high-contrast (full) and dimmer low-contrast (red-filtered) lighting conditions. In experiment one, zeaxanthin-supplemented birds had significantly increased retinal carotenoid levels, but declined in foraging performance in the high-contrast condition relative to astaxanthin-supplemented birds that showed no change in retinal carotenoid accumulation. In experiments one and two combined, we found that retinal carotenoid concentrations predicted relative foraging performance in the low- vs. high-contrast light conditions in a curvilinear pattern. Performance was positively correlated with retinal carotenoid accumulation among birds with low to medium levels of accumulation (∼0.5–1.5 µg/retina), but declined among birds with very high levels (>2.0 µg/retina).

**Conclusion/Significance:**

Our results suggest that carotenoid-mediated spectral filtering enhances color discrimination, but that this improvement is traded off against a reduction in sensitivity that can compromise visual discrimination. Thus, retinal carotenoid levels may be optimized to meet the visual demands of specific behavioral tasks and light environments.

## Introduction

Food detection is a major selective pressure shaping the visual systems of animals, and a primary goal of visual ecologists is to understand the links between the environment, foraging behavior, and the physiology and function of the visual system [1]. For example, the evolution of trichromatic color vision in primates is thought to be driven by selection for the detection of red fruits against green foliage [2], and the spectral sensitivities of numerous aquatic species are precisely matched to the light spectra available in their habitats [3]. Natural selection on the visual system, in the foraging context, can subsequently shape sexually selected signals in animals through the process of sensory drive [4]. By favoring signals matched to the sensitivities of the visual system, sensory drive can lead to the evolution of elaborate coloration and the emergence of new species (e.g [5]).

Foraging may also have a much more direct influence on the performance of the visual system because it determines the availability of nutrients necessary for the development, maintenance, and function of the eye. For example, retinal (or vitamin A aldehyde) is an essential component of the photopigments of all animals and must be acquired from food, and diet-derived carotenoid pigments act as intraocular filters to protect the eye and tune spectral sensitivities of photoreceptors in many species [6]. Therefore, the visual capabilities of an individual may not only be shaped by natural selection for the ability to find food on an evolutionary time scale, but also the quality and quantity of that food consumed within the individual's lifetime.

**Figure 1:**
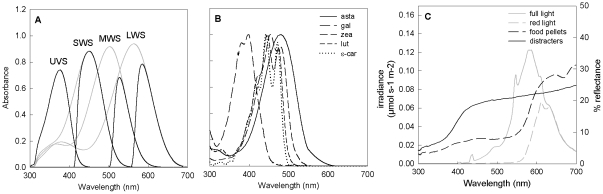
A comparison of carotenoid and visual pigment absorbance spectra, food and background reflectance, and irradiance spectra of the experimental lighting. (A) Absorbance spectra of single-cone photoreceptors before (gray lines) and after (black lines) carotenoid-pigmented cone oil-droplet filtering. Spectral sensitivities are based upon measures from the canary (*Serinus canaria*; [28]), the house finch's closest relative for which these values are known. Microspectorphotometric studies [10] suggest that the long-wavelength-sensitive cone (LWS) is filtered by an oil droplet pigmented with astaxanthin, the medium-wavelength-sensitive cone (MWS) is filtered by a zeaxanthin-pigmented oil droplet, the short-wavelength-sensitive cone (SWS) is filtered by a galloxanthin-containing oil droplet, and the ultraviolet-sensitive cone (UVS) has a transparent oil droplet. (B) Normalized absorbance spectra of carotenoids found in the house finch retina: astaxanthin (asta), galloxanthin (gal), zeaxanthin (zea), lutein (lut), and ε-carotene (ε-car). (C) Sample irradiance spectra from the full and red-filtered room lights and reflectance spectra of the food pellets and distracters. Irradiance spectra are presented in gray and are associated with the y-axis on the left. Reflectance spectra are presented in black and associated with the y-axis on the right.

Among vertebrates, birds have some of the most complex and capable visual systems and are a model for the study of visual ecology [7]. Avian color vision is based upon the response of four types of single-cone photoreceptors that range in sensitivity from the ultraviolet through the entire human-visible spectrum (Fig. 1a, [8]). A separate class of long-wavelength-sensitive double cones is thought to be responsible for achromatic (luminance) discrimination [9,9], and scotopic (i.e. low-light) vision depends upon rod photoreceptors. Carotenoids accumulate within the cone photoreceptors in oil droplets located between the inner and outer segments [10] and filter the light reaching the visual pigment. The types and concentrations of carotenoids in the oil droplets are specific to the cone types (Fig. 1b, [10]) and thus act as matched filters that enhance color discrimination, improve color constancy, provide photoprotection, but also reduce the quantum catch of the photoreceptor (Fig. 1a, [11]).

Carotenoids are particularly interesting components of the visual system because their accumulation 1) is dependent upon environmental availability and acquisition, and 2) may be traded off among multiple functions in the body, including antioxidant protection, immune system performance, and body coloration [12]. Birds cannot produce carotenoid pigments *de novo*, but must acquire them through their diet, and carotenoid accumulation in the retina is sensitive to recent dietary pigment intake [13], as well as, immune system activation [14]. These shifts in retinal carotenoid accumulation have the potential to alter cone oil-droplet filtering and visual performance [15]. Recently, Knott et al. [16] examined the influence of dietary carotenoid supplementation on cone oil droplet filtering of zebra finches (*Taeniopygia guttata)* and crimson rosellas (*Platycercus elegans*) and observed subtle shifts in the absorbance of specific types of oil droplets in specific regions of the retina. They concluded that these small changes were unlikely to affect spectral sensitivity; however this was not tested directly.

In this study, we examined the influence of dietary carotenoid supplementation and retinal carotenoid accumulation on the visually mediated foraging behavior of the house finch (*Carpodacus mexicanus*). The house finch is a common North American passerine and a model species for the study of sexual selection and the evolution of elaborate ornaments [17]. Male finches display sexually selected carotenoid-based plumage coloration that varies from drab yellow to deep red, depending upon dietary carotenoid access and health [17], and we have found that retinal carotenoid accumulation follows much the same pattern as plumage carotenoids. For example, retinal carotenoid levels are positively correlated with body condition and plumage coloration [18], immune challenges deplete carotenoids from the retina [14], and levels of some carotenoid types (e.g. galloxanthin) are dependent upon dietary carotenoid intake [13]. Color vision plays an important role in foraging in this species, as house finches actively discriminate among food items based upon color [19], [20]. Therefore, if changes in retinal carotenoid accumulation alter color vision, they may also impact visual foraging behavior.

In our first experiment, we tested this hypothesis by measuring the foraging performance of captive finches before and after supplementing them with dietary carotenoids. We tested foraging by presenting birds with red-dyed food items in a matrix of achromatically variable inedible distracters under two lighting conditions that produced high or low chromatic contrast conditions with similar levels of achromatic contrast. We predicted that dietary carotenoid supplementation would enhance carotenoid-mediated spectral tuning in retina, thereby improving food detection and foraging. Specifically, we predicted that carotenoid-supplemented birds would find more food items in both lighting conditions and that the difference in foraging performance between the high- and low-contrast lighting conditions would diminish following supplementation as compared to the low-carotenoid birds. We also examined the influence of carotenoid supplementation on food color preferences by measuring the consumption of sunflower seeds dyed various colors [20], with the prediction that carotenoid supplementation would improve discrimination and strengthen existing color preferences.

Because dietary supplementation has a relatively limited effect on the accumulation of retinal carotenoids [13], we included data from a second experiment and took a correlational approach to investigate the relationship between direct measures of retinal carotenoid accumulation and visual foraging performance. We predicted that the relative number of food items eaten in the low- vs. high-contrast condition would be positively correlated with direct measures of retinal carotenoid accumulation.

## Methods

### Ethics statement

All experiments were carried out under United States Fish and Wildlife Service permit #MB088806-1 and Arizona State Game and Fish scientific collecting permit SP727468. All experimental procedures were approved by the Institutional Animal Care and Use Committee at Arizona State University (protocol #09-1054R and 06-874R).

### Study animals and carotenoid supplementation

#### Experiment 1

In June 2009, we captured 14 adult male and 14 adult female house finches on the campus of Arizona State University in Tempe, Arizona, USA in baited basket traps (for details see Toomey and McGraw [18]). We housed the birds individually in small wire cages (0.6 m×0.4 m ×0.3 m) in two greenhouse rooms with *ad libitum* access to tap water and a very low carotenoid (0.078±0.031 µg/g) base diet of sunflower seeds. The greenhouse was illuminated with sunlight, and throughout the study the birds were maintained on a natural photoperiod. The birds were fed the base diet for eight weeks to minimize retinal carotenoid variation stemming from dietary differences in the wild. In weeks seven and eight of the initial depletion period, we tested foraging performance (see below) and in week nine we randomly assigned birds to one of three diet treatments: 1) control – four males and four females received the base diet and tap water with a non-carotenoid vitamin supplement (Vita-Sol®, United Pet Group EIO, Tampa, FL); 2) zeaxanthin – five males and five females received a supplement of zeaxanthin beadlets (35 µg/ml of OptiSharp® DSM, Heerlen, Netherlands) suspended in their drinking water and the vitamin supplement; and 3) astaxanthin - five males and five females received a supplement of astaxanthin beadlets (35 µg/ml of Carophyll Pink® DSM, Heerlen, Netherlands) suspended in their drinking water and the vitamin supplement. The birds were given the supplements *ad libitum* each weekday for eight weeks (weeks 9–16), with plain tap water provided on weekends. At the start of week 17 and continuing through week 18, all birds were returned to the base seed and tap-water diet and we again tested foraging performance (see below). Carotenoids deplete from the retina relatively slowly compared to other tissues, requiring ≥4 weeks of deprivation to cause significant declines [13]; thus this final depletion period was an effort to decouple any immediate effects that carotenoid supplementation might have on health state (and perhaps foraging motivation) from the effects of carotenoid accumulation in the retina. At end of 18 weeks, we euthanized all birds and collected retinas to directly measure carotenoid accumulation (see below).

#### Experiment 2

In November 2009, we captured and housed 27 female house finches to study the influence of dietary carotenoid supplementation on female mate choice behavior (data not presented here). We trapped these finches as described in experiment one and maintained them on a sunflower seed diet. In January 2010, we randomly selected 13 females and supplemented their drinking water with carotenoids (zeaxanthin: 17.5 µg ml^−1^ OptiSharp® DSM, Heerlen, Netherlands), while the remaining 14 birds continued on the unsupplemented sunflower seed diet. Supplementation continued for eight to ten weeks and, following a depletion period as described in experiment one, we tested the foraging performance of all birds (see below). At the conclusion of the mate choice tests, we euthanized all birds and collected retinas to directly measure carotenoid accumulation (see below).

### Foraging performance test

We developed a foraging task based upon the methods of Caine and Mundy [21] and Maddocks et al. [22], in which birds were challenged to pick out food pellets from a visually contrasting matrix. Although more precise behavioral tests of color vision are available (e.g. [23]), we chose this method because it offers three advantages: 1) it does not require extensive training and can be rapidly learned by wild birds, 2) it is easily scaled to test a relatively large number of individuals and, 3) this task is analogous to ground foraging for seeds, the primary mode of foraging in the house finch [24].

We presented each bird with 30 rice pellets (3.5 mm diam.; Careline rice diet, Roudybush, Woodland, CA) dyed with red food coloring (McCormick & Company Inc., Sparks, MD; Fig. 1c, Fig. S1) in a matrix of inedible distracters varying from tan to black of similar shape and size as the food pellets (Kaytee Soft-Sorbent, Kaytee Products Inc. Chilton, WI). The food pellets and distracters were presented on white paper plates (15.3 cm diam.) in the housing cage of each bird, with water, but not food, available throughout each trial. In experiment one, the birds were tested three times under two lighting conditions before (weeks 9–10) and after (weeks 17–18) carotenoid supplementation. In experiment two, the birds were tested only after dietary supplementation (weeks 17–20). All trials lasted 20 min. and were carried out only once per day and began at 0800 hrs following overnight food deprivation, to ensure that birds were motivated to forage. After each trial, we collected plates, recovered any spilled pellets and distracters, and counted the number of food pellets remaining as a measure of foraging performance. The number of pellets eaten in each of the three trials was moderately repeatable within individuals (*R* = 0.578; [25]), and for subsequent analyses we calculated mean of the three repeated trials in each lighting condition at each time point. In experiment one, we investigated possible diet- and lighting-condition effects on the activity levels of the birds, by video recording the foraging behavior of a subset of birds (4/treatment group) in both lighting conditions during the post-supplementation period. From these videos, we measured the amount of time the birds spent actively foraging.

Foraging tests were carried out in a windowless indoor room under two lighting conditions: (1) full, unfiltered fluorescent light (Sylvania, 34W, T12 rapid start Super Saver, Osram-Sylvania, Danvers, MA, USA), or (2) red-filtered-light created by placing filters (Roscolux Fire #19, Rosco Laboratories Inc., Stamford, CT, USA) over the fluorescent lights (Fig. 1c, Fig. S1). The filters were set up the night before the trials at ∼1800 hrs, to allow the birds time to acclimate to the new conditions. To assess how lighting conditions affected food-pellet conspicuousness, we measured 15 reflectance spectra from the food pellets and distracters, as well as three irradiance spectra of the filtered and unfiltered-light, using an Ocean Optics USB2000 spectrophotometer (Ocean Optics Inc., Dunedin, FL, USA; Methods S1). We then used the noise-limited receptor model [23],[26],[27], with the spectral sensitivities of the Canary (*Serinus canaria*, a cardueline-finch relative of house finches [28]), to calculate the chromatic and achromatic contrasts between the food pellets and distracters and among the distracters under both lighting conditions (electronic supplementary material). These measures confirmed that the food items contrasted significantly with the background distracters and that this contrast differed between the lighting conditions (Table 1). Specifically the chromatic contrast of the food items against the background distracters was significantly greater than the contrast among the distracters, while the achromatic contrast was not significantly different between food and background distracters compared to the contrast among the distracters (Table 1). To estimate the effects of the relatively dim light conditions in our experiment, we also calculated the visual contrasts with an estimate of photon noise for dim environments [29]. The inclusion of photon noise in the model reduced the magnitude of the contrasts but did not alter the pattern of contrast between food and distracters relative to the contrast among the distracters (Table 1).

**Table 1:** Total irradiance and predicted visual contrasts between food pellets and background distracters under experimental illumination modeled assuming either bright or dim (photon-noise limited) conditions.

Lighting	Total irradiance (µmol s^−1^ m^−2^)	Vision model	Contrast between food and distracters (jnds) ± st. dev.		Contrast within distracters (jnds) ± st. dev.	
			chromatica,c	achromatic^b^	chromatic^c^	achromatic
Full	12.92±6.47	bright	21.41±6.22	9.66±6.48	4.39±3.26	8.93±6.86
		dim	5.32±1.52	2.44±1.67	1.03±0.75	2.46±1.76
Red	5.10±1.94	bright	19.86±5.58	7.56±5.63	5.40±4.08	9.16±7.01
		dim	2.94±0.83	1.45±1.07	0.99±0.76	1.89±1.35

a >1 jnd difference between lighting conditions (*p*<0.001) for both vision models.

b >1 jnd difference between lighting conditions (*p* = 0.007) for bright vision model only.

c >1 jnd difference between food/distracter contrast and distracter/distracter contrast (*p*<0.001) in both vision models.

### Food color preference test

In experiment one, prior to the second foraging performance test (week 16), we measured the food color preferences of all birds following the methods of Bascuñán et al. [20], with the following modifications to match the timing and duration of the foraging performance tests. The test began at 0800 hrs, lasted 20 min., and 20 of each red, green, yellow and orange dyed sunflower seeds were presented on the same paper plates used in the foraging performance tests. However, no distracters were present during the food color preferences tests, and the tests were carried out under the semi-natural lighting conditions of the greenhouse housing room. We measured the number of seeds of each color eaten by counting the seeds remaining at the end of the trial.

### Carotenoid analyses

We quantified amounts of specific carotenoid types in the left retina of each bird using high performance liquid chromatography (HPLC). Extraction procedures, analytical methods, and the results of experiment 1 are reported in Toomey & McGraw [13].

### Statistical analyses

Analyses were carried out in SPSS13 (SPSS inc., Chicago, IL), and values are reported as mean ± SE throughout. To examine the influence of lighting conditions on the number of food pellets eaten, we used repeated-measures analyses of variance (rmANOVA), with the number of food pellets eaten in each lighting condition as the within-subjects factor and sex as a between-subjects factor. Because the number of pellets eaten differed significantly between lighting conditions (§3b), we tested the effects of dietary carotenoid supplementation on foraging performance in separate rmANOVAs for full and red-light, with the number of pellets eaten before and after supplementation as within-subjects factors and sex and supplementation treatment as between-subjects factors. Food color preferences were tested using rmANOVA, with seed color as the within-subjects factor and sex and supplementation treatment as the between-subjects factors. Non-significant interaction terms were removed from the models, Greenhouse-Geisser corrections were used when the models deviated from the assumptions of sphericity, and the significance level was set to α = 0.05.

To test the relationship between our direct measures of retinal carotenoid accumulation and changes in foraging performance, we carried out separate repeated-measures analyses of covariance (rmANCOVA), with the number of food pellets eaten before and after supplementation as the repeated measure, sex as a between-subjects factor, and total retina carotenoid concentration as a covariate, under each lighting condition. Concentrations of all six retinal carotenoid types were significantly intercorrelated [13], but because they are associated with different photoreceptors [10] they may influence visual function in different ways. To explore the individual association between each of the six different retinal carotenoid types and the change in foraging performance, we calculated separate Pearson's correlations.

Because dietary supplementation had a relatively limited effect on the accumulation of retinal carotenoids [13], we took a correlational approach to further investigate the relationship between retinal carotenoid accumulation and visual foraging performance. We fitted linear and polynomial regressions of total retinal carotenoid concentration against the number of pellets eaten in the low-contrast, red-filtered-light condition relative to the high-contrast, full-light condition. We limited this analysis to the foraging tests in the post-diet-manipulation period of experiments one and two, just prior to taking our direct measures of retinal carotenoids.

## Results

### Dietary supplementation and retinal carotenoid accumulation

#### Experiment 1

The effects of dietary supplementation on retinal carotenoid accumulation are reported elsewhere (Experiment 3 in Toomey and McGraw [13]). To summarize, birds supplemented with zeaxanthin had significantly higher levels of retinal galloxanthin and ε-carotene than birds receiving the astaxanthin and control diets. There were no significant differences in the accumulation of any retinal carotenoids between the astaxanthin-supplemented and control birds. Carotenoid supplementation did not significantly affect accumulation of astaxanthin, zeaxanthin, or lutein in the retina, and there were no significant sex differences in retinal carotenoid accumulation.

#### Experiment 2

**Figure 2:**
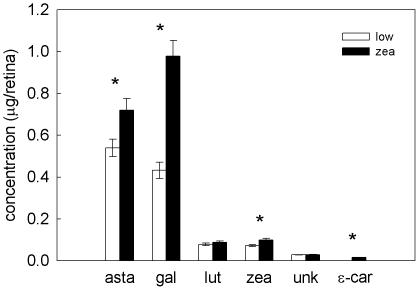
Retinal carotenoid levels in experiment two. Mean ± S.E. concentration of the six carotenoid types in retinas of female finches receiving a low-carotenoid (open bars) or zeaxanthin-supplemented (solid bars) diet in experiment two. *indicate significant treatment differences.

Female finches receiving the zeaxanthin-supplemented diet had significantly higher retinal carotenoid levels than females maintained on the low-carotenoid diet (Wilks' λ = 0.29, *F*_6,20_ = 7.89, *p* = 0.00018, Fig. 2). Specifically, retinal astaxanthin, galloxanthin, zeaxanthin, and ε-carotene levels were significantly higher in the high-carotenoid treatment (*F*_1,25_ = 6.90, *p* = 0.014, *F*_1,25_ = 43.40, *p*<0.0001, *F*_1,25_ = 9.71, *p* = 0.0046, *F*_1,25_ = 10.51, *p* = 0.0033 respectively). All retinal carotenoid types were significantly positively intercorrelated (*r*>0.40, p<0.037), with the exception of galloxanthin and an unidentified carotenoid (*r* = 0.30, *p* = 0.13).

### Effects of lighting conditions on foraging performance

#### Experiment 1

**Figure 3:**
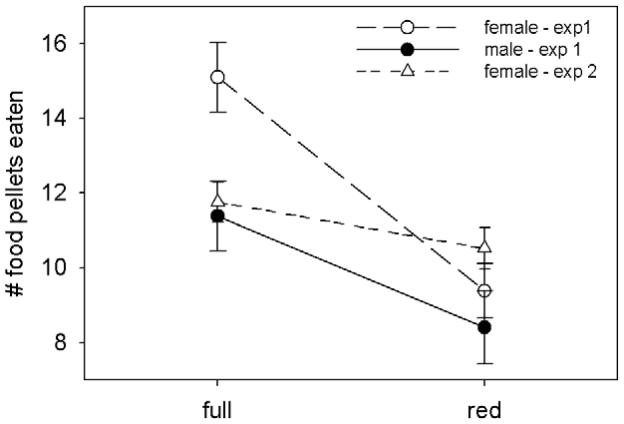
Foraging performance under experimental lighting conditions. Mean ± S.E. number of food pellets eaten by male and female house finches in experiment one, and by female house finches in experiment two under high-contrast full-light vs. low-contrast red-light conditions.

Prior to carotenoid supplementation, birds ate significantly fewer food pellets in the low-contrast, red-filtered-lighting condition than in unfiltered full-light (rmANOVA lighting: *F*_1,24_ = 49.24, *p*<0.0001, Fig. 3). This effect was stronger for females than males (rmANOVA lighting × sex: *F*_1,24_ = 4.95, *p* = 0.036, Fig. 3). Prior to supplementation, treatment groups did not differ significantly in foraging performance in either lighting condition (rmANOVA lighting × treatment: *F*_1,24_ = 0.39, *p* = 0.68). The number of food pellets eaten in individual trials ranged from 0–24 under red light, and 3–27 under full light, and all individuals consumed pellets under each lighting condition in at least one of the three trials.

#### Experiment 2

Female finches ate significantly fewer food pellets in the low-contrast, red-filtered-lighting condition than in unfiltered full-light (rmANOVA lighting: *F*_1,25_ = 5.72, *p* = 0.025, Fig. 3).

### Effect of dietary carotenoid supplementation on foraging performance

**Figure 4:**
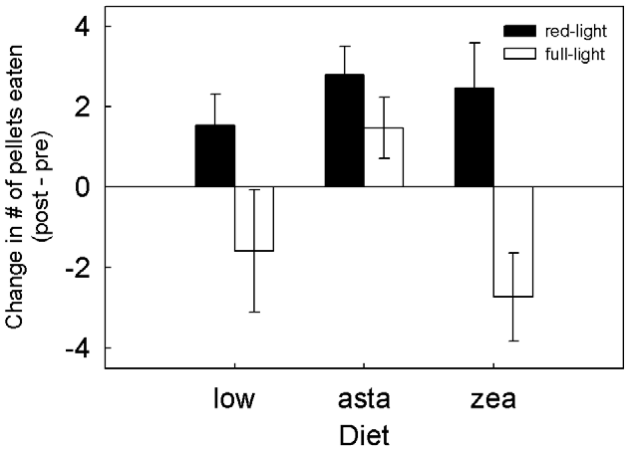
Carotenoid supplementation and foraging performance. Mean ± S.E. change in the number of food pellets eaten by finches in the red-filtered light (solid bars) and the full light (open bars) following eight weeks on a low carotenoid, astaxanthin- (asta) supplemented, or zeaxanthin- (zea) supplemented diet.

There was a significant effect of dietary carotenoid supplementation on number of food pellets eaten in the full-light condition (rmANOVA time × treatment: *F*_2,24_ = 5.25, *p* = 0.013, Fig. 4). The number of food pellets eaten by zeaxanthin-supplemented birds in full-light declined following supplementation and differed significantly from the astaxanthin-supplemented group (Tukey's post-hoc, *p* = 0.014), but not control birds (Tukey's post-hoc, *p* = 0.71). Supplementation had no significant effect on foraging in the red-light condition (rmANOVA time × treatment: *F*_2,24_ = 1.84, *p* = 0.620, Fig. 4). The change in the number of food pellets eaten in full-light differed significantly between the sexes (rmANOVA time × sex: *F*_1,24_ = 8.50, *p* = 0.008); females declined over time (pre: 15.00±0.93 vs. post: 12.4±0.95), while males remained relatively constant (pre: 11.3±0.93 vs. post: 12.0±0.95). There was a significant increase in the number of food items eaten in the red-filtered-light condition over time across all diet treatments (rmANOVA time: *F*_1,24_ = 18.92, *p*<0.0001, Fig. 4); this increase did not differ between the sexes (rmANOVA time × sex: *F*_1,24_ = 1.59, *p* = 0.22). In the subset of birds for which we observed behavior during the trials, the amount of time spent actively foraging did not differ significantly between lighting conditions (*F*_1,8_ = 0.59, *p* = 0.47), the sexes (*F*_1,8_ = 0.027, *p* = 0.87), or among treatment groups (*F*_2,8_ = 2.88, *p* = 0.11). Over the course of these trials, we occasionally observed the birds making errors, picking up the distracters, manipulating them in their bills, and subsequently rejecting them.

Consistent with the treatment effects described above, retinal carotenoid levels, measured at the conclusion of experiment one, significantly predicted the change in the number of food pellets eaten in full-light before and after supplementation (total carotenoids: *F*_1,25_ = 5.19, *p* = 0.032). In separate analyses of the different retinal carotenoid types, concentrations of retinal galloxanthin and ε-carotene were significantly negatively correlated with the change in the number of food pellets eaten in full-light (*r = *−0.480, *p* = 0.014 and *r = *−0.435, *p* = 0.021 respectively). Concentrations of other retinal carotenoid types were not significantly correlated with the decline in foraging performance (asta: *r* = −0.377, lut: *r* = −0.138, zea: *r* = −0.329, unk: *r* = −0.163). The temporal improvement in foraging performance in red-filtered-light was not significantly related to retinal carotenoid accumulation (*F*_1,25_ = 0.78, *p* = 0.387).

### Carotenoid supplementation and food color preference

**Figure 5:**
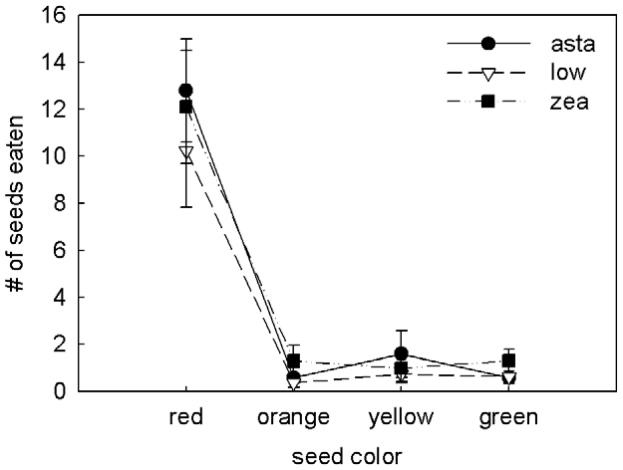
Food color preferences of carotenoid-supplemented birds. Mean ± S.E. number of seeds dyed each of four colors eaten during the 20 min food preference trial. Diet treatments are denoted with different symbols.

Seed consumption differed significantly by color type (*F*_1.19,26.14_ = 56.17, *p*<0.0001), with finches eating significantly more red dyed seeds than all other colors (Tukey post-hoc test, p<0.0001; Fig. 5). Food color preferences did not differ between the sexes (*F*_1.18,26.14_ = 0.21, *p* = 0.694). There was no significant effect of dietary carotenoid supplementation on seed color preference (*F*_2.37,26.14_ = 0.25, *p* = 0.813) or on the total amount of food eaten (*F*_2,22_ = 0.71, *p* = 0.502).

### Retinal carotenoid accumulation and foraging performance in high vs. low contrast conditions

#### Experiment 1

**Figure 6:**
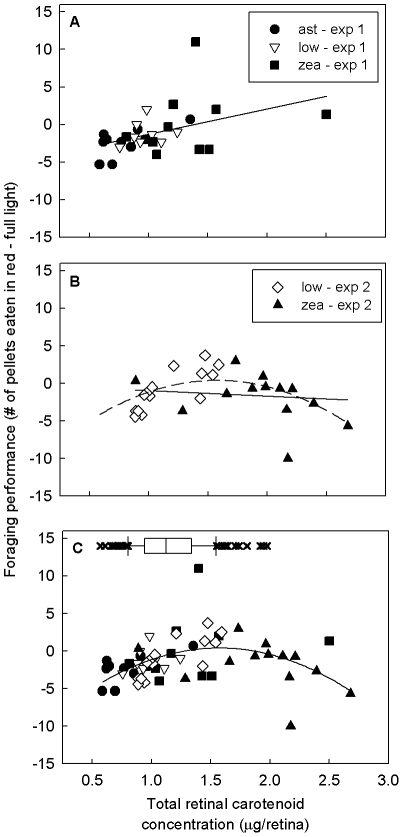
Retinal carotenoid levels and low contrast foraging performance. Relative number of food pellets eaten in the low-contrast red-light, as compared to high-contrast full-light, in the post-supplementation period for (A) experiment one, (B) experiment two, and (C) experiments one and two combined. The diet treatments within experiments one and two are denoted with different symbols. The box plot in at the top of figure C represents the natural range of variation in retinal carotenoid levels among wild house finches reported by Toomey and McGraw [18].

Foraging performance, measured as the relative number of pellets eaten in the low- vs. high- contrast condition, in the post-supplementation period did not differ significantly among diet treatments or between the sexes (*F*_2,24_ = 1.93, *p* = 0.17 and *F*_1,24_ = 2.83, *p* = 0.11 respectively). However, across sexes and treatment groups, total retinal carotenoid concentration was a significant positive predictor of relative foraging performance in the low-contrast condition (*r* ^2^ = 0.19, *F*_1,26_ = 5.92, *p* = 0.022, Fig. 6a). The correlation between retinal carotenoid accumulation and foraging performance was not specifically driven by our experiment-induced decline in foraging performance in the high-contrast condition (§3c). When we removed zeaxanthin-supplemented birds from the analysis, total retinal carotenoid concentration remained significantly positively correlated with foraging performance (*r* ^2^ = 0.34, *F*_1,26_ = 8.06, *p* = 0.012).

#### Experiment 2

Foraging performance did not differ significantly between diet treatments (*F*_1,25_ = 0.97, *p* = 0.33). There was no significant linear relationship between retinal carotenoid accumulation and foraging performance (*r* ^2^ = 0.017, *F*_1,26_ = 0.45, *p* = 0.51). However, an inspection of the plotted data suggested that the relationship was not linear; we therefore fitted a quadratic relationship between retinal accumulation and foraging performance and found a significant fit (*r* ^2^ = 0.34, *F*_2,24_ = 6.28, *p* = 0.006, Fig. 6b). The difference in relationship between retinal accumulation and performance observed in experiments 1 and 2 are likely the result of differences in the range of retinal carotenoid concentrations. Individuals in experiment one had relatively low retinal carotenoid levels (<1.5 µg/retina) where the quadratic curve can be approximated by a line. When we combined data from both experiments, we found that the quadratic curve provided a good fit (*r* ^2^ = 0.21, *F*_2,24_ = 6.76, *p* = 0.002), with the peak at 1.58 µg/retina, which falls just outside the 90^th^ percentile of retinal carotenoid accumulation for wild birds (Fig. 6c).

## Discussion

Our study provides the first evidence linking retinal carotenoid accumulation to visually mediated foraging behavior. Contrary to our predictions, dietary carotenoid supplementation and the subsequent increase in retinal carotenoid accumulation did not improve the foraging performance of house finches. Rather, birds with experimentally elevated retinal carotenoid levels showed a significant decline in foraging in the high-contrast lighting condition, while all birds, regardless of diet treatment, improved in the low-contrast condition. Surprisingly, we found that direct measures of retinal carotenoid accumulation predicted relative foraging performance in low- vs. high-contrast conditions in a curvilinear manner, such that performance peaked at an intermediate level and declined as levels increased or decreased. Although unexpected, these results are consistent with a carotenoid-mediated trade-off between color discrimination and low-light sensitivity.

The diet-driven decline in foraging performance is consistent with putative effects of retinal accumulation on visual function. Carotenoid-pigmented cone oil droplets are predicted to enhance color discrimination [11], [29], but this enhancement comes at the cost of reduced quantum catch and the potential for increased photon noise [29]. In dim conditions, contrast sensitivity declines with the square root of light intensity [30], and increased carotenoid filtering essentially reduces the intensity of light reaching the photoreceptors. Increased receptor noise levels can significantly reduce chromatic discriminability [15] and thus could limit the detectability of food items. Direct measures of oil droplet absorbance, coupled with behavioral tests at varying light intensities, are now needed to clarify mechanisms underlying these changes in visual foraging performance.

Although the diet-related changes in foraging are consistent with a visual mechanism, we cannot rule out more general influences of diet and learning. Regardless of dietary treatment, all birds improved their foraging efficiency in the low-contrast red light condition, suggesting that the birds learned to discriminate food more effectively and/or use different cues. The significant difference in full-light foraging performance that arose between zeaxanthin- and astaxanthin-supplemented birds may be attributable to changes in foraging motivation. For example, dietary carotenoid availability has been shown to influence color-based foraging preferences of guppies (*Poecilia reticulata*, [31]) and may have altered the motivation of the birds in our study to feed on red food items. Additionally, astaxanthin-supplemented birds received this red-colored carotenoid in their drinking water and may have become accustomed to consuming red material, potentially increasing their motivation to feed on the red food items. However, we found no difference in food color preferences or foraging effort between the diet treatments. We also observed significant differences in foraging behavior between the sexes over time, suggesting that foraging behavior is influenced by sex-specific physiological changes (experiment 1 included the transition from the breeding to molt period). Thus, we are left with an intriguing pattern, but further studies are needed to address these confounding factors and clarify the links between dietary carotenoids, retinal carotenoids, and visual foraging behavior.

Despite the unresolved relationship between dietary carotenoid supplementation and visual foraging performance, we found a significant relationship between direct measures of retinal carotenoid accumulation and visual foraging performance. Contrary to our prediction, performance was not linearly related to retinal carotenoid accumulation, but rather retinal levels predicted performance in a curvilinear manner, with peak performance at a carotenoid concentration of 1.59 µg retina^−1^. This peak is within the natural range of retinal carotenoid accumulation in house finches, falling near the 90^th^ percentile of wild birds examined in an earlier study [18], suggesting that performance may be optimized at a specific retinal carotenoid level. Optimization is consistent with a trade-off between chromatic discrimination and sensitivity that has been hypothesized for cone oil droplet filtering [29]. Under the relatively dim conditions of our low-contrast treatment, moderate levels of carotenoid accumulation may promote color discrimination, but high levels of accumulation may compromise discrimination by reducing photon catch and increasing photon noise (see above). Because photon noise levels depend upon the intensity of light [30], the carotenoid level, at which the costs and benefits of accumulation are balanced, should increase with increasing light intensities and this trade-off may disappear at high intensities. Although the light intensities used in this study are low compared to the natural, desert habitats of the house finch, they are comparable to conditions found under dense forest canopies [32]. An important next step will be to explore this trade-off in visual performance across the broad range of natural light intensities and among species that inhabit diverse light environments.

A carotenoid-mediated trade-off in avian visual function is supported by patterns of retinal carotenoid accumulation observed among species and individuals reared under varying light intensity. The retinas of nocturnal species (e.g. owls) have relatively pale oil droplets that presumably contain lower concentrations of carotenoids, which is hypothesized to improve their visual sensitivity under low light conditions [8]. In chickens (*Gallus gallus*), retinal carotenoid accumulation is developmentally plastic in response to light environment, such that chicks reared in dim environments develop less absorbent oil droplets with presumably lower carotenoid levels [33]. Thus, the demands of dim light vision may set a functional upper limit on the accumulation of carotenoids in the avian retina. Interestingly, very few (<10%) wild house finches exceed the “optimal” retinal carotenoid level identified in this study [18], yet we were able to push captive birds beyond this point with dietary supplementation. This suggests that the mechanisms of retinal accumulation are tuned to natural dietary carotenoid availability and/or birds use cues not available in captivity to regulate accumulation.

Linking visual foraging performance to retinal carotenoid accumulation is particularly intriguing because carotenoid-based male plumage coloration plays an important role in house finch mate choice [17]. Among wild house finches, we have found that retinal carotenoid levels are significantly positively correlated with male plumage redness [18], suggesting a potentially unique link between a sexually selected signal and the sensory system. Although dietary carotenoid supplementation [13] and immune system challenges [14] can cause small changes in retinal accumulation, much of the variation we have observed among wild birds remains unexplained. If retinal carotenoid accumulation is developmentally or genetically determined, then it could be linked with plumage color through common heritable variation in the mechanisms of carotenoid uptake and metabolism (e.g. lipoprotein production [34]). Alternatively, foraging could environmentally link vision and color signal expression, if vision-mediated food choice affects development of ornamental color. House finches have distinct food color preferences [19], [20] and may use color to select carotenoid- and/or antioxidant-rich foods (e.g. desert cactus fruits). Fruit color, for example, is a reliable indicator of antioxidant content (but not necessarily carotenoid levels [35]), and the increased consumption of antioxidants can enhance the expression of carotenoid-based colors [36], [37]. However, our results indicate that benefits of retinal carotenoid accumulation are not monotonic, and understanding their adaptive value will require a better understanding of the light environments in which foraging and mate choice occur.

The visual pigment sensitivities of birds are considered to be highly conserved among species [38], which has led to the widespread application of avian visual models based upon a relatively limited set of physiological parameters (e.g. [39]). Our results indicate that, within a species, visual discrimination can vary considerably in response to the physiological state of the eye. This complicates the interpretation of visual modeling results because discrimination may be influenced by the interacting effects of individual- and species-specific differences in retinal carotenoid accumulation with light intensity. This could be a particularly important consideration when assessing signaling and crypsis in dim environments, such as with colorful eggs and nestling mouths in cavity nests (e.g. [40]).

The trade-off between chromatic and luminance detection is an important force shaping the evolution of the visual system [9], [41], [42]. To date, visual ecologists have focused on how the genetically determined photoreceptor diversity and opsin-based spectral tuning mediate this trade-off [9], [41], [42]. However, our results suggest that inter-ocular filters (retinal carotenoids) mediate a similar trade-off in avian vision, opening up a range of new questions. Because retinal carotenoid accumulation is sensitive to alterations in diet, health, and developmental light environment [13], [14], [33], visual performance may also be shaped by the environment, not just over the course of generations, but throughout an individual's lifetime.

## Supporting Information

Figure S1 **Images of experimental food and lighting conditions.** (A) A sample image of the red food pellets and inedible gray paper distracters presented to the birds; (B) Unfiltered full-lighting conditions in our study room (left panel) compared to the red-filtered-lighting conditions (right panel).
(DOC)

Methods S1 **A detailed description of the methods used to measure the irradiance of the room lights and food and background spectral reflectance and the calculations of avian visual contrasts.**
(DOC)
