# Supplementary material for: The Effects of Dietary Carotenoid Supplementation and Retinal Carotenoid Accumulation on Vision-Mediated Foraging in the House Finch
Source: PLoS One. 2011 Jun 29;6(6):e21653. doi: 10.1371/journal.pone.0021653 (PMC3126843; doi:10.1371/journal.pone.0021653)

**Toomey and McGraw: Retinal carotenoids and visual foraging**

**Figure S1.** (A) A sample image of the red food pellets and inedible gray paper distracters presented to the birds; (B) Unfiltered full-lighting conditions in our study room (left panel) compared to the red-filtered-lighting conditions (right panel).

**A**


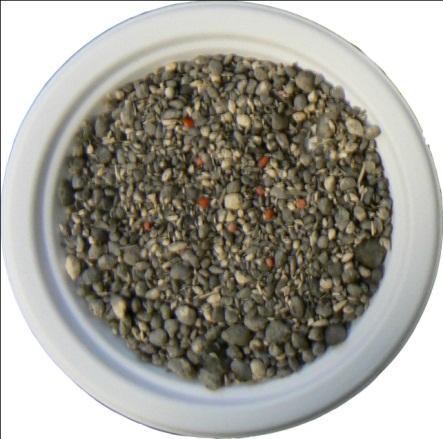


**B**


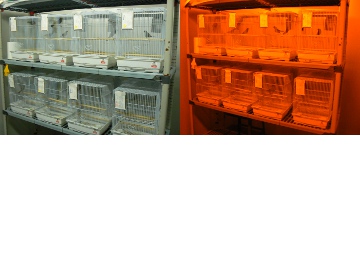

Supplement: Figure S1 — Images of experimental food and lighting conditions. (A) A sample image of the red food pellets and inedible gray paper distracters presented to the birds; (B) Unfiltered full-lighting conditions in our study room (left panel) compared to the red-filtered-lighting conditions (right panel). (DOC) [file pone.0021653.s001.doc]
