# Supplementary material for: The Effects of Dietary Carotenoid Supplementation and Retinal Carotenoid Accumulation on Vision-Mediated Foraging in the House Finch
Source: PLoS One. 2011 Jun 29;6(6):e21653. doi: 10.1371/journal.pone.0021653 (PMC3126843; doi:10.1371/journal.pone.0021653)
Supplement: Methods S1 — A detailed description of the methods used to measure the irradiance of the room lights and food and background spectral reflectance and the calculations of avian visual contrasts. (DOC) [file pone.0021653.s002.doc]

**Methods S1. The effects of dietary carotenoid supplementation and retinal carotenoid accumulation on vision-mediated foraging in the house finch**

**Reflectance spectra measurement:**

We measured reflectance spectra of food pellets and background distracter matrix, relative to a white standard (Spectralon; Labsphere Inc., North Sutton, NH, USA), with an Ocean Optics USB2000 spectrophotometer with a PX-2 pulsed xenon light source (Ocean Optics Incorporated, Dunedin, Florida, USA). Fifteen spectra each were collected from the food pellets and background distracters at coincident-normal geometry at a distance of 1 cm from the surface. The reflectance spectra were than binned to 1 nm intervals for subsequent analyses.

**Irradiance spectra measurement:**

We measured irradiance spectra under the two lighting conditions (full and red-filtered) using an Ocean Optics USB2000 spectrophotometer with a single fiber-optic probe (P400-1-UV-VIS; Ocean Optics) and a cosine-correcter with a 180° acceptance angle and a measurement surface of 6 mm in diameter (CC-3-UV; Ocean Optics). We calibrated the spectrophotometer with a standard light source (LS-1-CAL; Ocean Optics) and measured the downwelling irradiance spectra three times under each lighting condition within the housing cages of the birds.

**Chromatic contrast calculation:**

To calculate the avian visual system chromatic contrast between the food pellets and distracters, we followed the models proposed by Vorobyev et al. (1998) as modified by Aviles et al. (2008). We calculated the photoreceptor quantum catch for each cone class with the following equation:


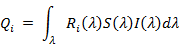


where λ indicates wavelength, *Q_i_* is the quantum catch for the *ith* photoreceptor, *R_i_(λ)* is the spectral sensitivity of the *ith* photoreceptor, *S(λ)* is the reflectance spectrum of the color patch, and *I(λ)* is the irradiance spectrum. We calculated photoreceptor sensitivity based on physiological data from the canary detailed below (Table S2). To assess the influence of lighting conditions on chromatic contrast, we repeated these calculations using the irradiance spectra from either full or filtered red-light.

We then calculated the log ratio of the quantum catches for the food pellets against the background distracters:


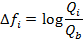


where *Q_b_* is calculated with the reflectance spectrum of the background distracters.

We calculated the chromatic contrast in just-noticeable differences as follows:


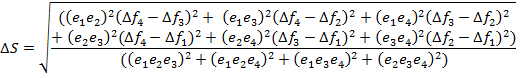


To account for the potential effects of the relatively dim conditions in our experiment, we calculated the light intensity independent noise (e_i_) for a given photoreceptor following Vorobyev et al. (1998)


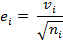


and light intensity dependent noise (e_i_) following Osorio et al. (2004)


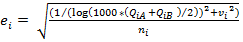


*Q_iA_* and *Q_iB_* are the quantum catches of the ith photoreceptor for the food items and background relative to a 100% reflecting surface.  *v_i_* is the noise in a single photoreceptor that we set to 0.05 in our model and *n_i_* is the number of receptors of type *i*, which are given in table S2.

**Achromatic contrast calculation:**

We modeled avian perception of the achromatic contrast between food pellets and distracters following Siddiqi et al. (2004) as presented by Loyau et al. (2007). We calculated the quantum catch of the double-cone photoreceptors as follows:


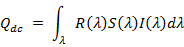


We then calculated the log ratio of the quantum catches for the food pellets and background distracters:


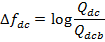


where *Q_dcb_* is calculated with the reflectance spectrum of the background distracters.

We calculated the achromatic contrast as follows:


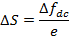


where the receptor noise *e* is calculated as follows:


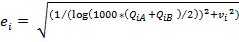


**Modeling spectral sensitivity:**

We modeled the absorbance spectra of the photoreceptors based upon the λ_max_ values reported by Hart and Vorobyev (2005) given in table S2 below and the visual pigment template of Govardovskii et al. (2000) as follows:


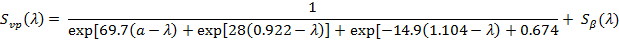


where:


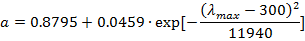


and S_β_(λ) equals the absorbance of the β-band of the opsin absorbance spectrum:


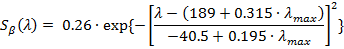


To account for the spectral tuning of the cone oil droplets, we used the cut-off values given in table S2 and model template proposed by Hart and Vorobyev (2005) as follows:


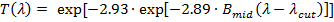


For all models we used the ocular media transmission (*T_ocular_*(λ)) of the starling (Hart et al. 1998). Therefore, spectral sensitivity for a given photoreceptor was defined as:


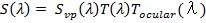


**Table S2. Visual system parameters based upon the canary (*Serinus canaria;* Das et al. 1999)**

| parameter | value |
| --- | --- |
| Cone oil droplet - λ_cut_ and (*B_mid_)* |  |
| P-type* | 413 (0.095) |
| C-type | 414 (0.095) |
| Y-type | 506 (0.054) |
| R-type | 578 (0.054) |
| Visual pigment (λ_max_) |  |
| UVS/VS | 363 |
| SWS | 440 |
| MWS | 501 |
| LWS | 567 |
| Cone ratios (*u,s,m,l*) | 1,1,2,2 |
|  |  |

*P-type oil droplet parameters were not available for the canary; therefore we used values from the blue tit (Hart et al. 2000). Published *b_mid_*values are not available for the P-type oil droplet; therefore we set this to the same value as the C-type oil droplet.

**References**

**Das D, Wilkie SE, Hunt DM, Bowmaker JK**1999. Visual pigments and oil droplets in the retina of a passerine bird, the canary *Serinus canaria*: microspectrophotometry and opsin sequences. *Vision Research* 39:2801-2815

**Govardovskii, V. I., Fyhrquist, N., Reuter, T., Kuzmin, D. G., & Donner, K.**2000. In search of the visual pigment template. *Visual Neuroscience,*17, 509-528.

**Hart, N. S., Partridge, J. C., & Cuthill, I. C.**1998. Visual pigments, oil droplets and cone photoreceptor distribution in the European starling (*Sturnus vulgaris*). *Journal of experimental biology,*201, 1433-1446.

**Hart, N. S., Partridge, J. C., Cuthill, I. C., & Bennett, A. T. D.**2000. Visual pigments, oil droplets, ocular media and cone photoreceptor distribution in two species of passerine bird: the blue tit (*Parus caeruleus* L.) and the blackbird (*Turdus merula* L.). *Journal of comparative physiology A: Neuroethology, sensory, neural, and behavioral physiology,*186, 375-387.

**Hart, N. S. & Vorobyev, M.**2005. Modelling oil droplet absorption spectra and spectral sensitivities of bird cone photoreceptors. *Journal of Comparative Physiology A-neuroethology sensory neural and behavioral physiology,*191, 381-392.

**Hart, N. S.**2002. Vision in the peafowl (Aves: *Pavo cristatus*). *Journal of Experimental biology,*205, 3925-3935.

**Vorobyev, M., Osorio, D., Bennett, A. T. D., Marshall, N. J. & Cuthill, I. C.**1998. Tetrachromacy, oil droplets and bird plumage colors. *Journal of comparative physiology A: Neuroethology, sensory, neural, and behavioral physiology,* 183, 621-633.

**Osorio, D., Smith, A., Vorobyev, M. & Buchanan‐Smith, H.  .**2004. Detection of Fruit and the Selection of Primate Visual Pigments for Color Vision. The American Naturalist, 164, pp. 696-708

**Siddiqi, A., Cronin, T. W., Loew, E. R., Vorobyev, M., & Summers, K.**2004. Interspecific and intraspecific views of color signals in the strawberry poison frog *Dendrobates pumilio*. *Journal of experimental biology,*207, 2471-2485.
